# 

**Published:** 1850-03

**Authors:** 


### NOTICE TO CORRESPONDENTS.

Communications and Books for notice should be addressed to the Editor, care of Messrs. Lindsay & Blakiston.

Letters, &c., connected with the *business affairs* of the Journal should be addressed to the Publishers.

Papers for publication must be received *before* the 20th of the month, or they cannot appear in the forthcoming number.

Several of our domestic exchanges have either come very late to hand, or have entirely failed to reach us. Those only that have been received since Feb. 1st are included in the following list.

The Boston Medical and Surgical Journal. (Weekly, Boston.)
Western Journal of Medicine and Surgery. (Received late.)
Medical News.
Western Lancet. (Received late.)
The British American Journal of Medical and Physical Science. (Montreal.)
The London Lancet. (Weekly, London.)
The Medical Times. (Weekly, London.)

---

The foreign correspondents of the Examiner will please direct their Exchanges and other communications to the care of Mr. Charles J. Skeet, 27 King William St., Charing Cross, London, or Mr. H. Bossange, 21 Bis, Quai Voltaire, Paris.

